# A genetic correlation scan identifies blood proteins associated with bone mineral density

**DOI:** 10.1186/s12891-022-05453-z

**Published:** 2022-06-03

**Authors:** Jiawen Xu, Shaoyun Zhang, Haibo Si, Yi Zeng, Yuangang Wu, Yuan Liu, Mingyang Li, Limin Wu, Bin Shen

**Affiliations:** Orthopedic Research Institute, Department of Orthopedics, Sichuan University West China Hospital, 37# Guoxue Road, Chengdu, 610041 Sichuan Province PR China

**Keywords:** Bone mineral density, Linkage disequilibrium score regression, Human plasma protein, Genome-wide association study, Osteoporosis, Blood proteome

## Abstract

**Background:**

Osteoporosis is a common metabolic bone disease that is characterized by low bone mass. However, limited efforts have been made to explore the functional relevance of the blood proteome to bone mineral density across different life stages.

**Methods:**

Using genome-wide association study summary data of the blood proteome and two independent studies of bone mineral density, we conducted a genetic correlation scan of bone mineral density and the blood proteome. Linkage disequilibrium score regression analysis was conducted to assess genetic correlations between each of the 3283 plasma proteins and bone mineral density.

**Results:**

Linkage disequilibrium score regression identified 18 plasma proteins showing genetic correlation signals with bone mineral density in the TB-BMD cohort, such as MYOM2 (coefficient = 0.3755, *P* value = 0.0328) among subjects aged 0 ~ 15, POSTN (coefficient = − 0.5694, *P* value = 0.0192) among subjects aged 30 ~ 45 and PARK7 (coefficient = − 0.3613, *P* value = 0.0052) among subjects aged over 60.

**Conclusions:**

Our results identified multiple plasma proteins associated with bone mineral density and provided novel clues for revealing the functional relevance of plasma proteins to bone mineral density.

**Supplementary Information:**

The online version contains supplementary material available at 10.1186/s12891-022-05453-z.

## Introduction

Osteoporosis (OP) is a metabolic bone disease characterized by low bone mass. It is thought to be induced by an imbalance between the process of bone resorption and that of bone formation [1]. The typical symptom is the bone fragility fracture which often occurs in older individuals, especially women [2, 3]. It is estimated that more than 23 million men and women are at high risk of osteoporotic fractures in the European Union [3]. The probability of suffering any major osteoporotic fracture is approximately 20% for men and 50% for women after the 1950s [4].

Bone mineral density (BMD) indicates the amount of bone mineral in bone tissue. Low BMD is the typical pathological change of osteoporosis. Due to the different statuses of bone resorption and formation during human life, BMD varies throughout the whole life course. Body mass continues to increase during infancy and reaches the peak body mass (PBM) in the second or third decade of life [4, 5]. It gradually declines due to overwhelming bone resorption, which occurs with increasing age [4].

Human BMD and PBM can be affected by both environmental and genetic factors [6]. However, it seems that heredity exerts greater influence than the environment [7]. The heritability of BMD is thought to range from 60 to 85% [8]. Recent genome-wide association studies (GWAS) have identified a group of genes, such as GPC6 and SPTBN1, as new candidate determinants of BMD [9, 10]. Previous genetic studies also identified multiple proteins and blood metabolites related to BMD, such as BGH3 and 1,5-anhydroglucitol [11, 12]. Moreover, the physiological characteristics of bone vary in different growth stages, as does BMD. However, the specific genetic characteristics underlying the BMD variation in all life stages remain unclear.

Human plasma proteins, also known as blood proteins, include a substantial amount of proteins in the circulatory system [13]. These proteins are essential in various cell processes, such as mineral transportation and immunity defense. Because plasma proteins are critical to various physiological processes, some plasma proteins are recognized as good drug targets [13]. Moreover, facilitated by the development of proteomics, numerous plasma proteins have been found to be involved in a variety of pathological changes, which has led to the promotion of disease diagnosis strategies [14]. For example, Kim H et al. suggested that the human plasma DPP4 level is related to osteoporotic fracture and may help to evaluate the risk of fracture [15].

It has been demonstrated that genetic factors considerably contribute to the regulation of gene expression [16]. Extensive efforts have been made to explore the effect of DNA polymorphisms on gene expression levels and to identify many gene expression quantitative trait loci (eQTLs) [16]. However, because the above-mentioned approach is insufficient for unveiling the relationship between genetic variation and protein expression, the importance of identifying protein quantitative trait loci (pQTLs) has been gradually highlighted. Benjamin et al. analyzed more than 3000 plasma proteins and identified a group of protein pQTL loci in healthy subjects. This study is helpful for exploring the genetic relationships between plasma proteins and complex human diseases [13].

Genetic correlation is the part of variance that two or more traits share due to genetic factors. It is quite common in complex diseases. Linkage disequilibrium score regression (LDSC) is a powerful tool and is frequently used to accurately estimate genetic correlations among different diseases or traits based on the summary data of GWAS [17]. For example, a previous study conducted LDSC analysis and identified almost 300 genetic correlations among 24 traits including anorexia, obesity and educational attainment [18].

In this study, we conducted a large-scale scan of genetic correlation between BMD and human plasma proteins. LDSC analysis was conducted by integrating each GWAS summary dataset of 2 BMD cohorts and the protein pQTLs involving over 3000 human plasma proteins. The first cohort is a GWAS database of life-course total body BMD (TB-BMD), and the second is a GWAS database of BMD estimated by quantitative ultrasound of the heel (eBMD) [19, 20]. The proteins identified by both two cohorts were regarded as candidate proteins in this study. Our research provides novel clues for the etiological studies of BMD.

## Materials and methods

### GWAS summary data of the TB-BMD cohort

The GWAS summary data of life-course total body BMD (TB-BMD) were used here [19]. This study consists of 30 epidemiological cohorts of TB-BMD and includes 66,628 subjects from America, Europe, and Australia. Approximately 90% of the individuals are of European ancestry. SNP genotyping was conducted via the Affymetrix UK Biobank Axiom array/UK BiLEVE Axiom array, Omniexpress array and Illumina arrays. Genotype imputation was performed using the cosmopolitan (all ethnicities combined) 1000 Genomes phase 1 v.3 (March 2012) reference panel. In this study, all subjects were divided into five age groups, including 0 ~ 15 years, 15 ~ 30 years, 30 ~ 45 years, 45 ~ 60 years, and 60 or more years. Dual-energy X-ray absorptiometry (DXA) was used to measure the TB-BMD of the population above 15 years old. Total body less head (TBLH) was used in individuals younger than 15 years old. After adjusting for age, weight, height, genomic principal components (derived from GWAS data) and other study-specific covariates for BMD, linear regression models were built in each study among all 5 groups. The basic characteristics of the populations included in this meta-analysis is summarized in supplementary Table 1. Other details of the participants, genotyping, imputation, meta-analysis and quality control can be found in the published study [19].

### GWAS summary data of the eBMD cohort

GWAS summary data of eBMD were derived from the UK Biobank [20]. In this study, GWAS analysis was conducted in 426,824 UK Biobank full-release white British individuals (55% female). A total of 1103 conditionally independent signals were identified to be related to BMD at genome-wide significance (*p* < 6.6 × 10^− 9^). All of the participants in the UK Biobank were genotyped by the Affymetrix UK BiLEVE Axiom or Affymetrix UK Biobank Axiom array and centrally imputed by UK Biobank. For heel bone quality estimation, quantitative ultrasound speed of sound (SOS) and broadband ultrasound attenuation (BUA) quantitative ultrasound assessment of calcanei was performed using a Sahara Clinical Bone Sonometer [Hologic Corporation (Bedford, Massachusetts, USA)]. The basic characteristic of the populations included in this meta-analysis is summarized in Table 1. More information on the subjects, genotyping, imputation and statistics can be found in the previous study [20].

**Table 1:** The basic characteristics of the participants included in UK Biobank Study

**ENTIRE COHORT**		**FEMALES (*N*** **= 264,304)**						**MALES (*N*** **= 216,073)**					
**TRAIT**	**UNIT**	**MIN**	**MAX**	**RANGE**	**MEDIAN**	**MEAN**	**SD**	**MIN**	**MAX**	**RANGE**	**MEDIAN**	**MEAN**	**SD**
AGE	years	39.0	74.1	35.1	57.0	56.4	8.0	38.0	75.8	37.8	58.0	56.8	8.2
WEIGHT	kg	30.1	196.0	165.9	69.0	71.3	13.9	38.8	197.7	158.9	84.0	85.5	14.0
HEIGHT	cm	121.0	199.0	78.0	162.0	162.4	6.3	127.0	209.0	82.0	176.0	175.6	6.8
SOS	m/s^2^	1455.0	1696.6	241.6	1546.1	1549.0	30.3	1454.7	1707.9	253.2	1555.0	1558.0	31.5
BUA	dB/MHz	22.1	138.0	115.9	71.6	72.7	16.3	27.1	138.0	110.9	81.6	82.6	17.2
eBMD	g/cm^2^	0.15	1.02	0.87	0.51	0.52	0.12	0.18	1.05	0.87	0.55	0.57	0.12
**eBMD GWAS COHORT**		**FEMALES (*N*** **= 233,185)**						**MALES (*N*** **= 193,639)**					
**TRAIT**	**UNIT**	**MIN**	**MAX**	**RANGE**	**MEDIAN**	**MEAN**	**SD**	**MIN**	**MAX**	**RANGE**	**MEDIAN**	**MEAN**	**SD**
AGE	years	39.0	74.1	35.1	58.0	56.6	7.9	39.0	75.8	36.8	59.0	57.1	8.1
WEIGHT	kg	32.1	190.0	157.9	69.1	71.4	13.8	40.8	197.7	156.9	84.3	85.8	13.9
HEIGHT	cm	121.0	199.0	78.0	163.0	162.7	6.2	127.0	209.0	82.0	176.0	175.9	6.7
SOS	m/s^2^	1455.0	1696.6	241.6	1545.3	1548.2	29.9	1455.2	1707.7	252.5	1554.6	1557.6	31.3
BUA	dB/MHz	22.1	138.0	115.9	71.3	72.3	16.1	27.1	138.0	110.9	81.5	82.5	17.1
eBMD	g/cm^2^	0.15	0.99	0.85	0.50	0.51	0.11	0.18	1.05	0.87	0.55	0.56	0.12
**Fracture GWAS COHORT**		**FEMALE CASES (*N*** **= 31,709)**						**MALES CASES (*N*** **= 21,475)**					
**TRAIT**	**UNIT**	**MIN**	**MAX**	**RANGE**	**MEDIAN**	**MEAN**	**SD**	**MIN**	**MAX**	**RANGE**	**MEDIAN**	**MEAN**	**SD**
AGE	years	40.0	73.0	33.0	60.0	58.6	7.6	40.0	73.0	33.0	57.0	55.8	8.4
BMI		14.3	63.7	49.4	26.3	27.2	5.2	15.2	59.8	44.6	27.1	27.7	4.3
**Fracture GWAS COHORT**		**FEMALE CONTROLS (*N*** **= 201,444)**						**MALES CONTROLS (*N*** **= 172,167)**					
**TRAIT**	**UNIT**	**MIN**	**MAX**	**RANGE**	**MEDIAN**	**MEAN**	**SD**	**MIN**	**MAX**	**RANGE**	**MEDIAN**	**MEAN**	**SD**
AGE	years	39.0	71.0	32.0	57.0	56.4	8.0	39.0	73.0	34.0	59.0	57.1	8.1
BMI		12.1	69.0	56.9	26.0	27.0	5.1	12.8	68.4	55.6	27.3	27.8	4.1

*SOS* Speed of sound, *BUA* Broadband ultrasound attenuation, *eBMD* Estimated bone mineral density, *N* Sample size, *MIN* Minimum recorded value of trait, *MAX* Maximum recorded value of trait, *RANGE* Difference between maximum trait and minimum trait value, *MEDIAN* Median value of trait, *MEAN* Mean value of trait, *SD* Standard deviation

### Protein pQTL data of the human plasma proteome

The GWAS summary data of human plasma proteome was derived from the latest plasma proteome study [13]. Briefly, 1927 genetic associations (protein pQTLs) with 1478 proteins were identified in this study. A total of 3622 plasma proteins in 3301 subjects from the INTERVAL study were quantified through an expanded version of an aptamer-based multiplex protein assay (SOMAscan). A multiplexed, aptamer-based approach (SOMAscan assay) was utilized to measure the relative concentrations of the plasma proteins. Quality control was performed at both the sample and SOMAmer levels by controlling the aptamers and calibrator samples. The qualified samples were genotyped on the Affymetrix Axiom UK Biobank genotyping array at Affymetrix (Santa Clara, California, US). Variants were phased by SHAPEIT3 and successively imputed utilizing a combined 1000 Genomes Phase 3-UK10K reference panel via the Sanger Imputation Server (https://imputation.sanger.ac.uk). A total of 3283 plasma proteins were included in the GWAS summary data after quality control, which were utilized in the following genetic correlation analysis with BMD. More information on the participants, sample preparation and statistics can be found in the previous publication [13].

### Genetic correlation scan

In this study, we conducted a large-scale LDSC scan for potential genetic correlations between BMD and human blood proteins. Following the document of the LDSC tool (https://github.com/bulik/ldsc), the GWAS summary data of BMD and plasma proteins were prepared and input into LDSC for genetic correlation scanning using the default parameters recommended by the developers of LDSC. During LDSC analysis, the GWAS Z statistics of BMD and plasma proteins were derived from the GWAS and used as dependent variables for genetic correlation analysis [21]. In principle, LDSC analysis calculated each SNP’s ability to tag adjacent variants, denoted as the “LD score” [17]. A high LD score of SNP indicates that this SNP can tag more of other genetic variants, including causal sites [17]. As an efficient tool for assessing the genetic relationships among various complex traits and diseases, LDSC utilizes GWAS summary data rather than individual-level genotype data, which makes it more available and convenient [17].

## Results

### LDSC analysis results of the TB-BMD cohort

**Fig. 1:**
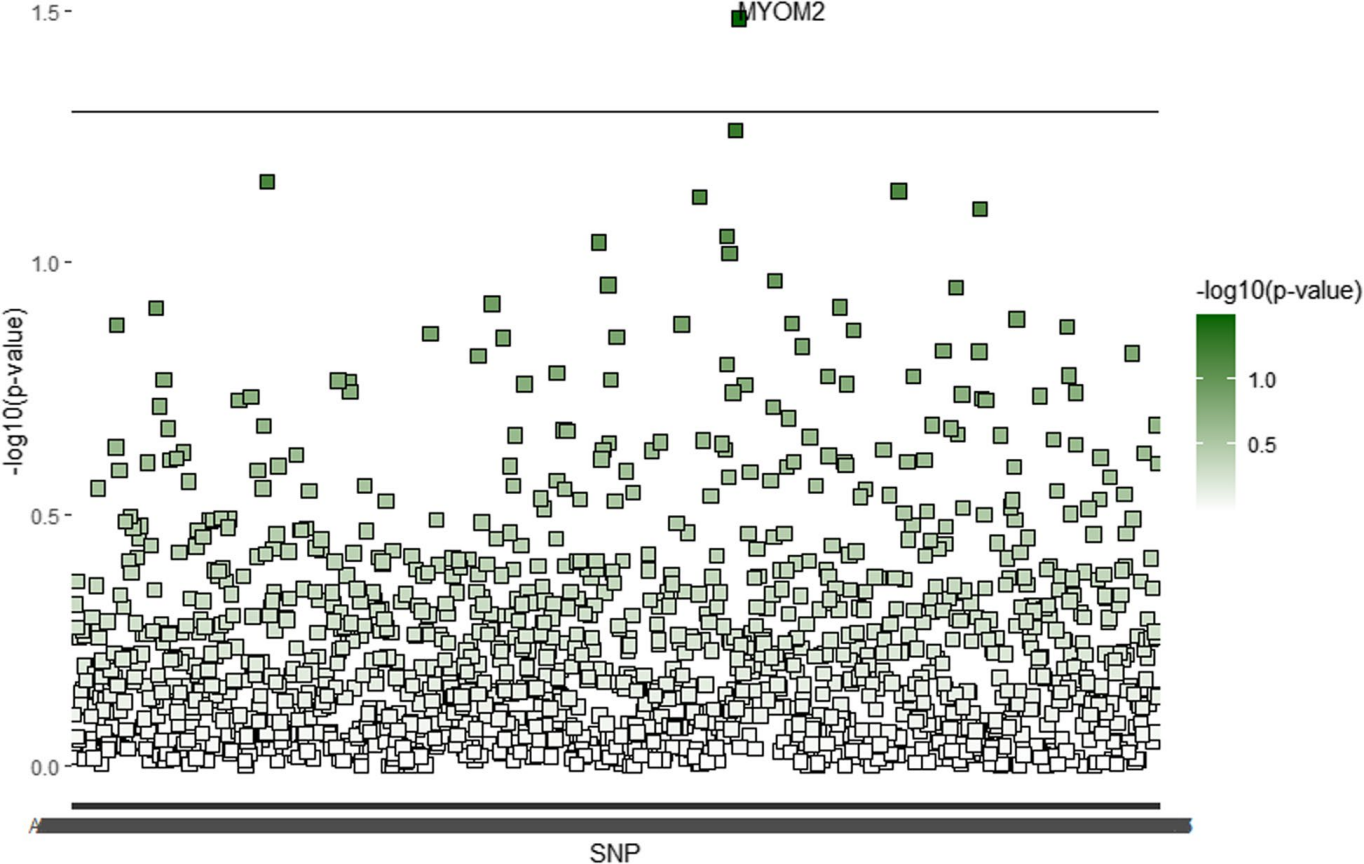
Human plasma proteins identified by linkage disequilibrium score regression for life course bone mineral density in subjects aged 0 ~ 15

**Fig. 2:**
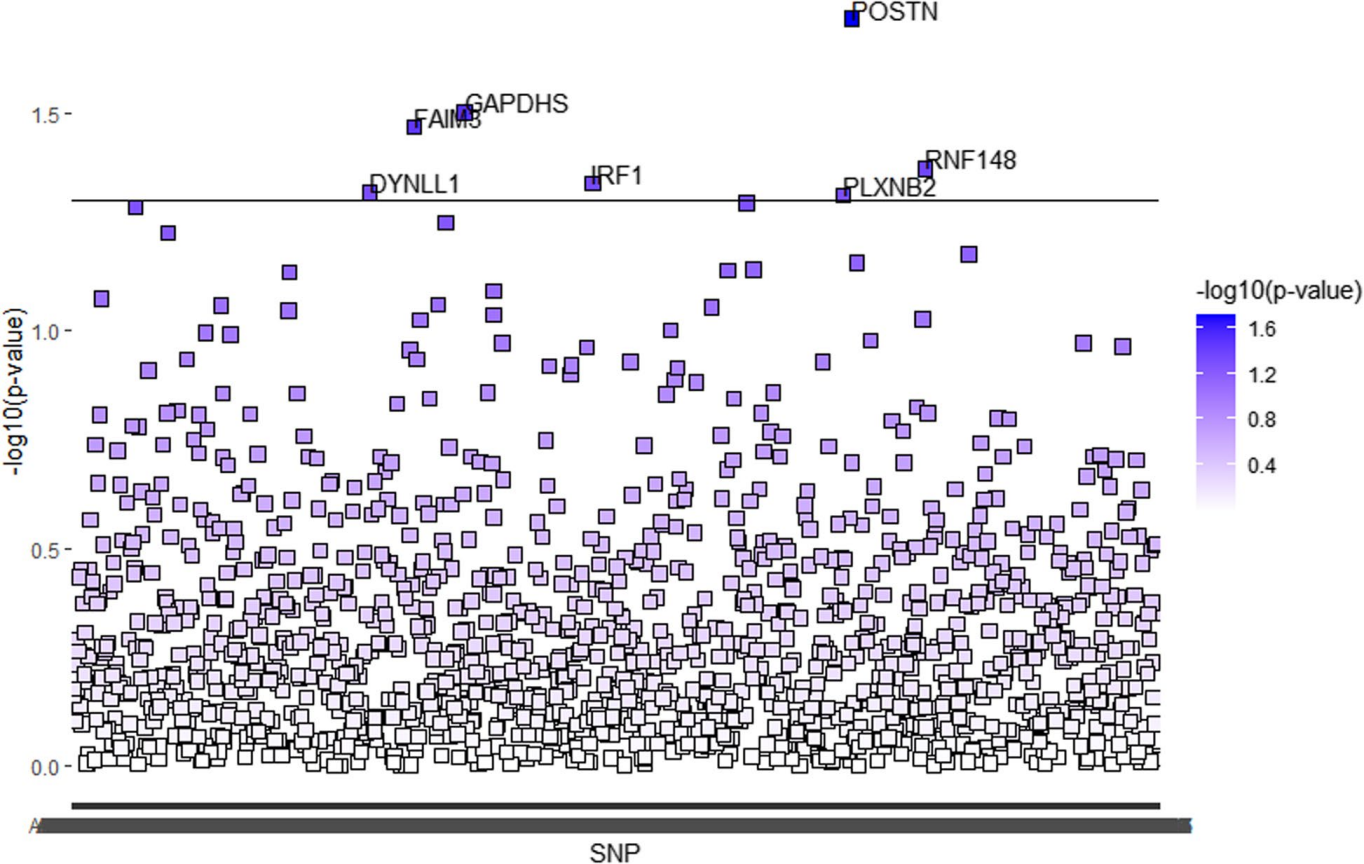
Human plasma proteins identified by linkage disequilibrium score regression for life course bone mineral density in subjects aged 30 ~ 45

**Fig. 3:**
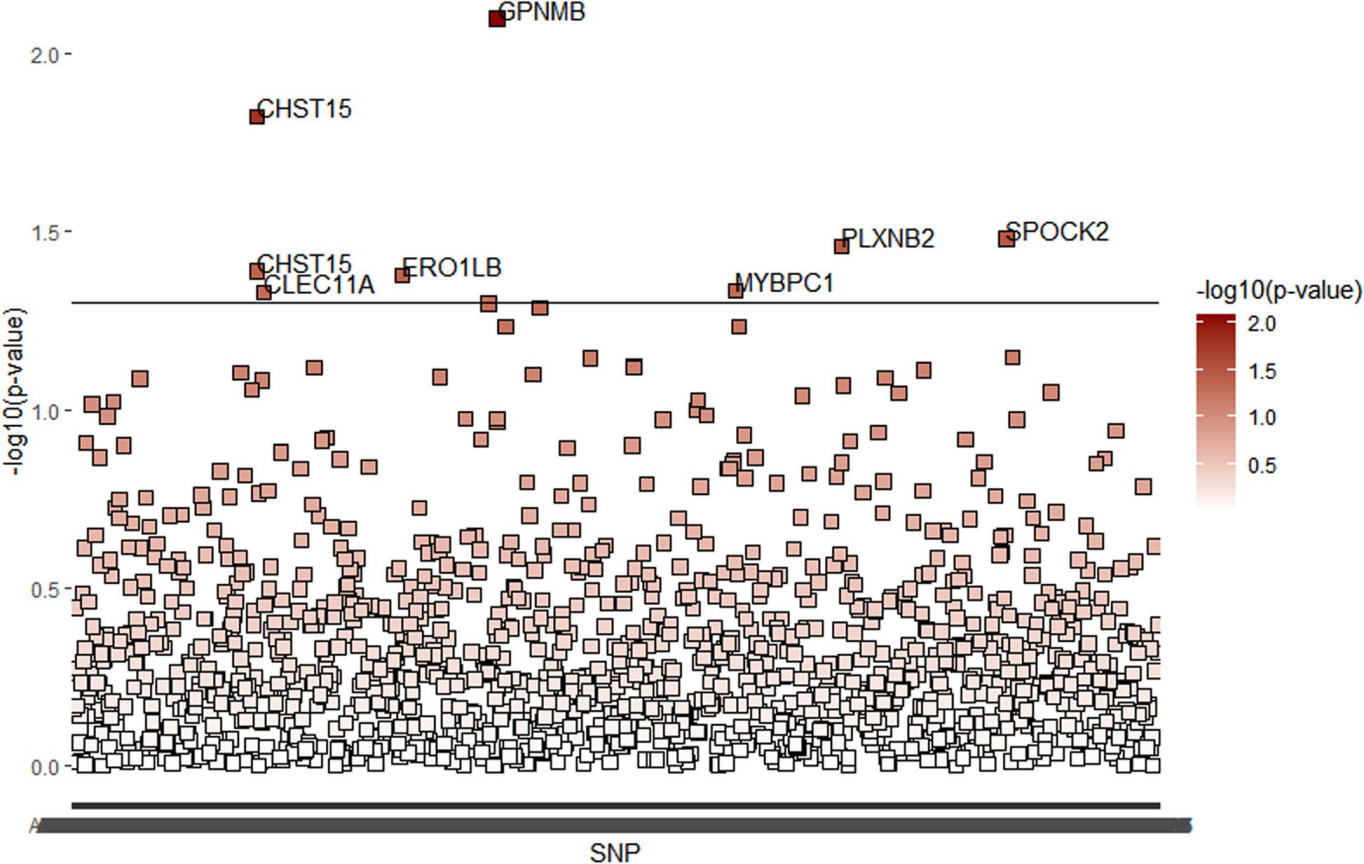
Human plasma proteins identified by linkage disequilibrium score regression for life course bone mineral density in subjects aged 45 ~ 60

**Fig. 4:**
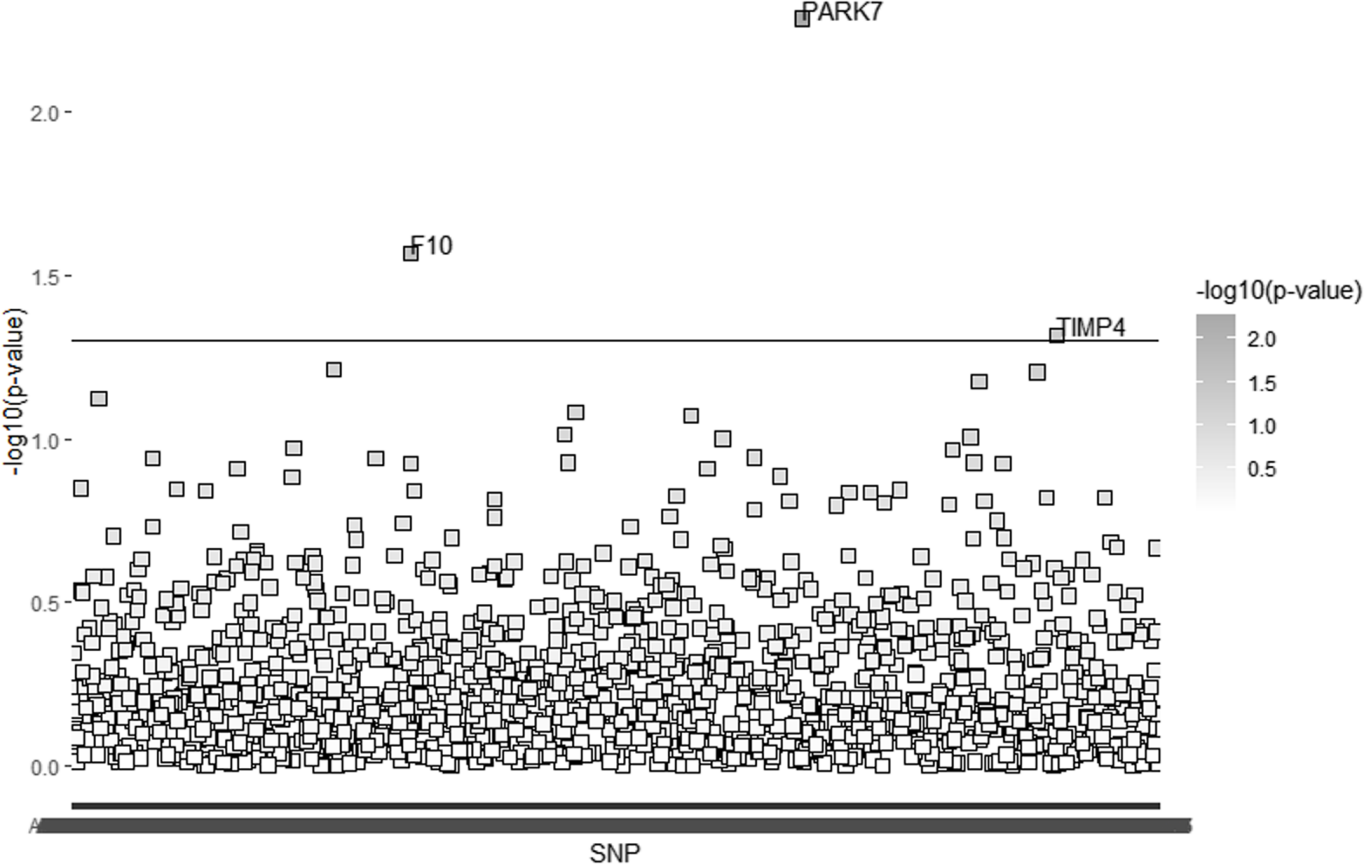
Human plasma proteins identified by linkage disequilibrium score regression for life course bone mineral density in subjects aged 60 or more years

For the TB-BMD cohort, LDSC analysis observed genetic correlations between 18 plasma proteins and life-course BMD (Table 2). In the group aged 0 ~ 15 years, MYOM2 was detected (coefficient = 0.3755, *P* value = 0.0328). No significant genetic correlations were observed between plasma proteins and the 15 ~ 30 aged group. In the group aged 30 ~ 45 years, 7 plasma proteins were identified such as periostin (coefficient = − 0.5694, *P* value = 0.0192) and G3PT (coefficient = 0.6272, *P* value = 0.0315). In the group aged 45 ~ 60 years, 8 plasma proteins were identified, including GPNMB (coefficient = 0.4921, *P* value = 0.008) and CHST15 (coefficient = 0.4835, *P* value = 0.0151). At ages greater than 60 years group, 3 plasma proteins were identified, including PARK7 (coefficient = − 0.3613, *P* value = 0.0052) and F10 (coefficient = − 0.4772, *P* value = 0.0271). Additionally, we found that PLXB2 appeared to be correlated with BMD in both the 30 ~ 45 years-old group (coefficient = 0.5184, *P* value = 0.0488) and the 45 ~ 60 years-old group (coefficient = 0.3927, *P* value = 0.0348) (Figs. 1, 2, 3 and 4).

**Table 2:** List of human plasma proteins identified by LDSC^a^ for Life course BMD^b^

Plasma protein	Gene	15 less age group		30 ~ 45 age group		45 ~ 60 age group		60 more age group	
		Coefficients	***P*** value	Coefficients	***P*** value	Coefficients	***P*** value	Coefficients	***P*** value
Myomesin-2	MYOM2	0.3755	0.0328	\	\	\	\	\	\
Periostin	POSTN	\	\	−0.5694	0.0192	\	\	\	\
Glyceraldehyde-3-phosphate Dehydrogenase, testis-specific	G3PT	\	\	0.6272	0.0315	\	\	\	\
Fas apoptotic inhibitory molecule 3	FAIM3	\	\	−0.3988	0.0341	\	\	\	\
RING finger protein 148	RN148	\	\	0.4332	0.0424	\	\	\	\
Interferon regulatory factor 1	IRF1	\	\	−0.4638	0.0459	\	\	\	\
Dynein light chain 1, cytoplasmic	DLC8	\	\	0.6325	0.0481	\\		\	\
Plexin-B2	PLXB2	\	\	0.5184	0.0488	0.3927	0.0348	\	\
Transmembrane glycoprotein NMB	GPNMB	\	\	\	\	0.4921	0.008	\	\
Carbohydrate sulfotransferase 15	CHST15	\	\	\	\	0.4835	0.0151	\	\
Testican-2	SPOCK2	\	\	\	\	0.3097	0.0332	\	\
ERO1-like protein beta	ERO1B	\	\	\	\	−0.3512	0.0419	\	\
Myosin-binding protein C, slow-type	MYBPC1	\	\	\	\	0.2941	0.0464	\	\
Stem Cell Growth Factor-beta	SCGF-beta	\	\	\	\	−0.4148	0.0468	\	\
Protein deglycase DJ-1	PARK7	\	\	\	\	\	\	−0.3613	0.0052
Coagulation Factor X	F10	\	\	\	\	\	\	−0.4772	0.0271
Metalloproteinase inhibitor 4	TIMP-4	\	\	\	\	\	\	−0.4097	0.0483

^a^Linkage disequilibrium score regression;

^b^Bone mineral density

### LDSC analysis results of the eBMD cohort

For the eBMD cohort, LDSC identified 21 plasma proteins for eBMD, such as RIPK2 (coefficient = − 0.2357, *P* value = 0.0072), RANTES (coefficient = − 0.2108, *P* value = 0.0145) and CRHBP (coefficient = 0.1533, *P* value = 0.0297) (Table 3).

**Table 3:** List of human plasma proteins identified by LDSC ^c^ for eBMD^d^ (*P* value < 0.05)

Plasma protein	Gene	Coefficients	***P***-value
Receptor-interacting serine/threonine-protein kinase 2	RIPK2	−0.2357	0.0072
C-X-C motif chemokine 16	CXCL16, soluble	0.2008	0.0119
C-C motif chemokine 5	RANTES	−0.2108	0.0145
Cathepsin Z	CATZ	0.1998	0.0178
Interleukin-7	IL-7	−0.2414	0.0212
Cysteine-rich hydrophobic domain-containing protein 2	CHIC2	0.1121	0.0256
Phenylalanine--tRNA ligase, mitochondrial	SYFM	0.2221	0.0256
Corticotropin-releasing factor-binding protein	CRHBP	0.1533	0.0297
O-acetyl-ADP-ribose deacetylase MACROD1	MACD1	0.1633	0.0319
Guanylate cyclase activator 2B	GUC2B	0.2065	0.033
SPARC-like protein 1	SPARCL1	0.1513	0.0362
Dickkopf-related protein 4	Dkk-4	−0.2058	0.0373
E3 ubiquitin-protein ligase ZNRF3	ZNRF3	0.1993	0.0419
Cellular retinoic acid-binding protein 1	RABP1	0.1496	0.0428
Cell growth regulator with EF hand domain protein 1	CGRE1	−0.1792	0.0436
Serine/threonine-protein kinase pim-1	PIM1	0.2707	0.0446
Creatine kinase M-type	CK-MM	0.2035	0.0471
Alpha-2-antiplasmin	a2-Antiplasmin	0.2072	0.0478
Tenascin-X	Tenascin-X	0.3227	0.0483
Thrombospondin-3	TSP3	0.2816	0.0488
Dickkopf-related protein 1	DKK1	−0.177	0.0495

^c^Linkage disequilibrium score regression

^d^Bone mineral density estimated by quantitative ultrasound of the heel

## Discussion

Altered plasma proteins have been found to be related to multiple human complex diseases. In this study, to explore the potential correlation between human plasma proteins and BMD across different life courses, we conducted a large-scale LDSC analysis by using two independent cohorts. We identified multiple plasma proteins correlated with BMD such as PARK7, SCGF-beta, POSTN, RANTES and GPNMB.

Recently, Lv et al. conducted a large-scale serum proteome analysis of OP and validated their results by comparing with the mRNA microarray quantitative analysis [22]. They identified several differentially expressed serum proteins with OP, such as POSTN [22]. Periostin, a secreted extracellular matrix protein in humans encoded by the POSTN gene, is also a significant protein identified by LDSC analysis in our study. It is expressed in many tissues, including the skeleton, and was originally identified in the periosteum and bone [23]. A previous study indicated that periostin participates in the early stages of osteoblast differentiation and bone formation [24]. It stimulates osteoblast functions and bone formation via integrin receptors and Wnt-beta-catenin pathways [25]. Experimental mice without periostin proportionately suffered from severe periodontal disease and bone density reduction [23]. Pepe J et al. suggested that serum periostin levels were associated with radial cortical porosity, even after adjusting for age [26]. Moreover, periostin expression declines with the skeletal growth, but it could be re-expressed in the process of fracture healing and bone repair [24]. It has also been demonstrated that it plays a key role in postmenopausal OP because serum levels can be measured to predict BMD and the risk of fracture [25]. These studies demonstrated the role of POSTN in the pathogenesis of OP.

Recently, Liu et al. evaluate the genetic correlation between plasma proteins and different sites of osteoarthritis (OA) by using LDSC analysis and they identified several suggestive plasma proteins with OA [27]. After comparing with the proteins identified for OA and our results, we found one overlapping protein, GPNMB showing genetic correlations with BMD of aged 45 ~ 60 group and hip OA. GPNMB, also known as osteoactivin, is a multi-functional transmembrane glycoprotein expressed in numerous tissues, including bone, osteoclasts and osteoblasts [28]. Abdelmagid et al. indicated that GPNMB depletion impaired osteoblast differentiation while overexpression of GPNMB facilitated osteoblast differentiation [29]. Other researchers have found that overexpression of GPNMB could induce transdifferentiation of C2C12 myoblasts into osteoblasts [30]. According to previous studies, the imbalance between the functioning of osteoclast and osteoblast cells leads to OP [31, 32]. While our findings may provide new insights into the role of GPNMB in the regulation of osteoclast and osteoblast cells and the pathogenesis of OA.

PARK7 (Parkinson disease protein 7, also known as protein deglycase DJ-1) belongs to the peptidase C56 family. According to a previous study, the PARK7/DJ-1 protein level was increased up to 3 times in MLO-Y4 osteocytic cells, which were treated with N-BPs (nitrogen-containing bisphosphonates), a kind of osteoporosis drug [33]. This change was demonstrated to be involved in a pathway that plays a role in the effect of N-BPs on osteocytes [33]. As reported by a recent study, short stature and brachydactyly are two characteristics observed in parkinsonism patients without the PARK7 region in the DJ-1 gene [33, 34]. The authors indicated that the PARK7 region may contain a modifier gene for bone growth [34]. These studies demonstrated that PARK7 may play an important role in bone growth.

SCGF-beta (Stem Cell Growth Factor-beta), also named Osteolectin or CLEC11A, was recognized as an osteogenic growth factor [35, 36]. Researchers have found that this protein promotes leptin receptor+ (LepR+) skeletal stem cells and other osteogenic progenitors in bone marrow to differentiate into osteoblasts and to maintain adult skeletal bone mass [36]. Andriani GA et al. indicated that CLEC11A is a component of SASP (senescence-associated secretory phenotype) [37]. They also suggested that aneuploid cells that accumulate during aging in some mammalian tissues potentially play key roles in age-related pathologies via SASP secretion [37]. These studies indicate that SCGF-beta have a direct pathogenic effect on bone metabolism. However, the relationship between SCGF-beta and BMD needs further research.

RANTES, also known as CCL5 (C-C motif chemokine ligand 5), is a chemokine gene clustered on the q-arm of chromosome 17 and is involved in immunoregulatory and inflammatory processes. By sharing common signaling pathways and regulatory mechanisms, the bone systems are closely related to the immune systems [38]. According to previous studies, CCL5 is directly associated with disturbed bone metabolism in nonpainful rheumatoid arthritis [38]. Additionally, the chemokine CCL5 is overexpressed in FDOJ (fatty oxide osteoporosis/osteolysis in the jawbone) cases [39]. Moreover, knowing that hyperhomocysteinemia is a risk factor for osteoporotic fractures, another previous study indicated that the protein CCL5 could be generated in osteoblasts after homocysteine induces serum amyloid A3 [38]. In summary, CCL5 may play an important role in bone metabolism, which needs more confirmative evidence.

Bone remodeling is a dynamic process, and BMD varies at different human life stages [19]. Because of bone reformation, BMD increases dramatically during childhood and adolescent periods, peaking at approximately the third decade of life [40]. Around the age of over 50, the process of bone resorption gradually overwhelms the process of bone reformation, which results in a decrease in BMD, particularly for postmenopausal women [41]. In this study, we identified several plasma proteins showing age-specific effects on BMD. For instance, coagulation Factor X showed a negative genetic correlation with BMD in subjects aged more than 60 years. Coagulation factor X is a vitamin K-dependent enzyme of the blood coagulation cascade and plays a critical role in blood coagulation [42]. Gigi R et al. demonstrated that rivaroxaban, an anticoagulant against factor Xa, could significantly induce a reduction in osteoblastic cell growth and energy metabolism and the inhibition of alkaline phosphatase, which is a kind of osteoblastic marker [43]. Coagulation factor X may contribute to the decrease in BMD of subjects aged more than 60 years by activating osteoblasts. Additionally, MYBPC1 appeared to be correlated with BMD in the subjects aged 45–60 years in this study. MYBPC1 encodes myosin binding protein c (slow type), which plays an important role in muscle contraction. MYBPC1 mutation has been linked to skeletal muscle atrophy-related disorders [44]. Furthermore, previous studies have demonstrated a positive association between body lean mass and BMD [45]. It is well known that adult body lean mass tends to decrease with age, especially after 40 years. Based on previous studies and our study results, we may infer that MYBPC1 contributes to the variation in BMD by affecting skeletal muscle loss in subjects aged 45–60 years.

However, we did not observe common plasma proteins in the two independent cohorts. This finding might indicate that the interactions between plasma proteins and different age groups or skeletal sites of BMD is highly specific. The previous study has identified variants in ESR1 and close proximity to RANKL showed a clear effect dependency on age for BMD [19]. According to recent studies, glucose homeostasis and anthropometric traits are associated with site-specific BMD and body mass index with age-specific BMD [46, 47]. Meanwhile, it has been reported that epigenetic modifications are highly dynamic, age-, and tissue-specific, and sensitive to endogenous signals and/or environmental stimulation [48]. Marini et al. suggested that epigenetics could represent a link between the genome and the environment to influence osteoporosis risk [48]. Those evidence together with our results may indicate that the combination of environmental factors and genetic variations contributing to the age-, and tissue-specific BMD.

There are some limitations that should be noted in this study. First, we used GWAS data obtained from European, American, Australian and multiethnic populations, in which most of the subjects were European. Because of the different genetic backgrounds of different populations, it is necessary to be careful when applying our study results to other populations, such as non-European. Especially for the group aged 0 ~ 15 years, there were more subjects of non-European ancestry than in the other groups. Second, the stability of the LDSC results can be influenced by the small sample size of the study. Meanwhile, the informative value of the obtained results is limited when using LDSC method only. Third, among the two datasets of BMD in our study, different body parts and different methods were used to measure BMD, which may have some effect on our results. Further efforts are still needed to confirm our results and clarify the potential biological mechanism underlying the observed genetic correlations between plasma proteins and BMD.

## Conclusions

To evaluate the genetic correlation between human plasma proteins and life-course BMD, we performed LDSC analysis in 2 cohorts. A number of human proteins were detected. Our results may provide new clues for the physiological process of human BMD and help improve the treatment of OP.

## Supplementary Information

**Additional file 1: Supplementary Table 1.** The basic characteristics of the participants included in per cohort and age strata.

## Data Availability

The large-scale LDSC scan for potential genetic correlations between BMD and human blood proteins was performed following the document of the LDSC tool (https://github.com/bulik/ldsc).

## Abbreviations

OP: Osteoporosis
BMD: Bone mineral density
PBM: Peak body mass
GWAS: Genome-wide association studies
eQTLs: Expression quantitative trait loci
pQTLs: Protein quantitative trait loci
LDSC: Linkage disequilibrium score regression
TB-BMD: Total body BMD
PARK7: Parkinson disease protein 7
SCGF-beta: Stem cell growth factor-beta
GAPDS: Glyceraldehyde-3-phosphate dehydrogenase
CCL5: C-C motif chemokine ligand 5
